# On the extreme hydrologic events determinants by means of Beta-Singh-Maddala reparameterization

**DOI:** 10.1038/s41598-022-19802-4

**Published:** 2022-09-15

**Authors:** Filippo Domma, Francesca Condino, Sara Franceschi, Davide Luciano De Luca, Daniela Biondi

**Affiliations:** 1 Department of Economics, Statistics and Finance ”Giovanni Anania”, University of Calabria, Arcavacata di Rende, CS Italy; 2 Department of Economics and Statistics, University of Siena, Siena, Italy; 3 Department of Informatics, Modelling, Electronics and System Engineering, University of Calabria, Arcavacata di Rende, CS Italy

**Keywords:** Environmental sciences, Hydrology, Natural hazards

## Abstract

In previous studies, beta-k distribution and distribution functions strongly related to that, have played important roles in representing extreme events. Among these distributions, the Beta-Singh-Maddala turned out to be adequate for modelling hydrological extreme events. Starting from this distribution, the aim of the paper is to express the model as a function of indexes of hydrological interest and simultaneously investigate on their dependence with a set of explanatory variables in such a way to explore on possible determinants of extreme hydrologic events. Finally, an application to a real hydrologic dataset is considered in order to show the potentiality of the proposed model in describing data and in understanding effects of covariates on frequently adopted hydrological indicators.

## Introduction

Over the last decades, growing attention have been addressed to the impact of hydrologic extreme events and to their possible relationship with climate change. It is indeed well known how the occurrence of extreme events, such as heavy rain, are responsible for a unduly large part of climate-related damages and hence are of great concern to the impact community and stakeholders^1,2^. The update and the improvement of useful models for better exploring observed extremes, with an emphasis on flood quantiles, are therefore strategic activities for the assessment of current and future exposure to risks. In this context, the hydrologists need for the most suitable model which not only gives rise to a good fit of data but is also based on realistic return level. The most used approach for modelling extreme events is conventional frequency analysis by adopting several common probability distributions such as the log Pearson type III^3^, the three-parameter Lognormal (^4^, pp. 208–238), the Generalized Pareto (see e.g.^4–6^, p. 615), the Generalized Logistic (see e.g.^7,8^), the Generalized Extreme Value (see e.g.^9,10^), the Generalized Gumbel (see e.g.^11,12^), the Two Component Extreme Value (TCEV^13^), and the Generalized Lindley^14^. Recently, more flexible distributions were proposed (see e.g.^15–17^): theoretically derived distributions of flood frequency account for the observed rainfall probability distribution and exploit rainfall-runoff models parameterized by means of geomorphological information (see e.g.^18–20^); other approaches represent non-asymptotic distributions for the annual maxima, and explicitly accounts for the random nature of the number of events/year and the inter-annual variability of the distributions of the ordinary events in each year^21,22^.

Moreover^23^, proposed the use of a new distribution function, namely four parameters Beta-Singh-Maddala distribution (so called because it is obtained by setting a parameter equal to 1 in the five parameters Beta-Singh-Maddala distribution), showing, by means of an application on real data regarding river flow maxima, its potentiality in extreme events analysis. A specific connection with two of the three special case of Generalized Extreme Value distribution has been proved, since this distribution belongs to the Fréchet maximal domain of attraction and to the Weibull minimal domain of attraction. With reference to the different techniques used in the literature to expand the families of distribution functions, we highlight that the four parameters Beta Singh-Maddala distribution corresponds to a proportional reversed hazard model or to a Lehmann type I distribution and can also be referred to as exponentiated Singh -Maddala distribution.

An additional common problem in hydrology is the estimation of flood quantiles in catchments having short data records or ungauged. Indeed accurate estimates of various streamflow statistics are crucial for water infrastructures design and for flood risk assessment, and they are routinely needed for ungauged catchments that lack nearby streamflow-gauged stations from which streamflow statistics could be directly computed. In this context, regional flood frequency analysis (RFFA) has been proposed in many regions worldwide on the basis of the concept that regional flood flow characteristics are closely related to basin and climate characteristics^24–32^. Moreover^33^, stated that regionalization should always be used in statistical analysis of extreme hydrological events because of the large influence that higher moments exert on the shape of the tail of the distribution which are focused by practical applications. Direct regression, geostatistical procedures, and index-flood method^34^ can be mentioned as example for RFFA^35^. In particular, the index-flood method coupled with L-moments^36^, has been extensively used worldwide^37–45^.

As^46^ emphasized, RFFA essentially consists of two principal steps: (i) identification of groups of hydrologically similar catchments, usually named homogeneous regions (HRs); (ii) development of prediction equations within each delineated region.

The identification of HRs often depends on subjective decisions^47^; traditionally, geographic and administrative boundaries have been used for defining homogeneous regions. Nevertheless, regions purely based on these characteristics may lack in hydrological homogeneity^48,49^. The method for identifying homogeneous regions in RFFA can be broadly divided in: (a) canonical correlation analysis, (b) cluster analysis, (c) hierarchical approach, (d) method of residuals, (e) region-of-influence (ROI), (f) canonical kriging and (g) flood seasonality regionalization (see e.g.^50–55^). As reported in^56^, cluster analysis can be considered as a state-of-art technique that can reduce the process subjectivity and regroup in a more appropriate way under a hydrological point of view. According to^57^, algorithms used for cluster analysis in regionalization studies can be categorized in hard (e.g., hierarchical, partitional, or hybrid) and fuzzy clustering. Moreover, there was a recent increase in the use of artificial intelligence (AI) and such techniques often provide superior results when compared with partitional clustering algorithms^54,58^. Whatever technique is adopted for HRs delineation, a critical point is the assessment of the plausibility of the obtained grouping and of the hypothesis of homogeneity for the proposed regions^59^. Moreover, the estimates are not smooth (both in geographic or physiographic space) due to possible discontinuities. Consequently, approaches that do not define fixed-boundary regions^60,61^ are receiving increasing attention: methods based on the interpolation of the hydrological variable in the descriptors space^24,62^, or based on the so-called top-kriging^63^.

As regards development of prediction equations, log-linear regressions techniques represent the most commonly used models. They allow to establish a relationship between hydrological variables and explanatory variables (such as drainage area, slope of the main channel, etc.). However, hydrological processes are naturally complex and consequently a simple log transformation could be insufficient for capturing this complexity. A recent work by^64^ compared the performances of several RFFA methods with respect to variable selection, variable transformation and delineation of regions. In particular they proposed the use of a generalized additive model (GAM) for dealing with nonlinearity between the dependent and predictor variables showing that, on the basis of their data, this approach generally outperforms the other methods even without linking GAM with a neighbourhood/region-of-influence approach.

Therefore, there is a huge literature on extreme hydrologic events concentrating on the modeling of extreme events and on regression techniques separately, while methods which simultaneously allow these evaluations seem to be less investigated. Some examples can be found in^65–67^ in the context of non-stationary series analysis. In this paper, we try to contribute by using the logic of the reparameterizations of the families of distribution functions and the one underlying the construction of the GAMLSS models. As known, the reparameterization techniques, when possible, allow to express the distribution function as a function of indicators of specific interest in the field of application, making it easier to interpret the behavior of the probability density function. The first contribution of this paper is to propose a new reparametrization of the Beta-Singh-Maddala, introduced by^23^, which allows us to express the distribution as a function of indicators of specific interest in the field of hydrological studies such as, for example, median and return level of hydrological variables. The second contribution of the paper consists in specifying regressive models for the dependence of the indicators on a set of explanatory variables using appropriate link functions in a similar way to what was done in the GAMLSS models. Consequently, the proposed method allows to overcome the fragmentation characterizing the generally used approach, that involves a first step aimed at indicator estimates (such as return level, mean, median) and a second step regarding regression on them.

## The Model

In this section, after a brief description of the Beta-Singh-Maddala distribution with four parameters (Beta-SM4), we present the general reparameterization assuming that there are four indicators, functions of the four parameters of Beta-SM4, of specific interest in the hydrological field and a set of explanatory covariates. Next, we study the particular case in which interest is placed on the median and on the return level.

### Reparameterization of the four parameters Beta-Singh-Maddala distribution

The four Beta-Singh-Maddala (Beta-SM4) has been proposed in the context of hydrologic data analysis by^23^, with the aim to properly describe some relevant aspects, such as the extent of return period and the amount and frequency of extreme values. Among the different properties demonstrated, we emphasize that the Beta-SM4 distribution turns out to be the distribution of the maximum of Singh-Maddala random variables, which belongs to the Frèchet maximal and to the Weibull minimal domain of attraction. Moreover, the authors highlight that this four-parameter distribution not only show a good overall fit on real data, but also a suitable representation of the extreme tails. Here, starting from this distribution, we propose its reparameterization, in order to make the parameters directly interpretable in terms of measures particularly relevant for hydrologic events description.

The Beta-SM4 distribution, in its original parameterization, has the following distribution function (*df*):

1 $$\begin{aligned} F_{Beta-SM4}(x;\varvec{\xi })=\left[ 1-\left( 1+\gamma _{2} x^{\gamma _{3}} \right) ^{-\gamma _{1}}\right] ^{a} \end{aligned}$$

where $\varvec{\xi }^{'}=(\gamma _{1},\gamma _{2},\gamma _{3},a )$, with $a>0$ and $\gamma _{k}>0$ for $k=1,2,3$. The probability density function (*pdf*) is given by

2 $$\begin{aligned} f_{Beta-SM4}(x;\varvec{\xi })= a\left[ F_{SM}(x;\varvec{\gamma }) \right] ^{a-1} f_{SM}(x;\varvec{\gamma }), \end{aligned}$$

where

3 $$\begin{aligned} F_{SM}(x;\varvec{\gamma })=1-\left( 1+\gamma _{2} x^{\gamma _{3}} \right) ^{-\gamma _{1}} \end{aligned}$$

and

4 $$\begin{aligned} f_{SM}(x;\varvec{\gamma })=\gamma _{1}\gamma _{2}\gamma _{3} x^{\gamma _{3}-1} \left( 1+\gamma _{2} x^{\gamma _{3}} \right) ^{-\gamma _{1}-1} \end{aligned}$$

are the *df* and *pdf* of Singh-Maddala distribution, where $\varvec{\gamma '}=(\gamma _1,\gamma _2,\gamma _3)$, see^68^.

From the expression of the *p*th quantile

5 $$\begin{aligned} x_{Beta-SM4}(p)=\gamma _{2}^{-\frac{1}{\gamma _3}} \left[ (1-p^{\frac{1}{a}})^{-\frac{1}{\gamma _1}} -1 \right] ^{\frac{1}{\gamma _3}} \end{aligned}$$

it is immediate to obtain median of Beta-SM4 distribution:

6 $$\begin{aligned} me_{Beta-SM4}(p)=\gamma _{2}^{-\frac{1}{\gamma _3}} \left[ (1-0.5^{\frac{1}{a}})^{-\frac{1}{\gamma _1}} -1 \right] ^{\frac{1}{\gamma _3}}. \end{aligned}$$

Furthermore, fixed the return period $\pi _{x_0}=[1-F_{Beta-SM4}(x_0;\varvec{\xi })]^{-1}$, the corresponding return level $x_0$ is given by

7 $$\begin{aligned} x_0(\pi _{x_0})=\gamma _{2}^{-\frac{1}{\gamma _3}} \left\{ \left[ 1-\left( 1-\frac{1}{\pi _{x_0}}\right) ^{\frac{1}{a}}\right] ^{-\frac{1}{\gamma _1}} -1 \right\} ^{\frac{1}{\gamma _3}}. \end{aligned}$$

For further properties and details on Beta-SM4 distrinution, see^23^.

Following the proposal of^69^, we consider the possibility of reformulate the Beta-SM4$(\gamma _1,\gamma _2, \gamma _3, a)$ in terms of new parameters, $I_j, j=1,...,4$, that are indicators describing some peculiarities of hydrologic data distribution and such that there exist a one-to-one transformation of the kind $I_j=g_j(\gamma _{1},\gamma _{2},\gamma _{3},a), j=1,...,4$, so that the system

8 $$\begin{aligned} {\left\{ \begin{array}{ll} I_1= g_{1}(\gamma _{1},\gamma _{2},\gamma _{3},a) \\ I_2= g_{2}(\gamma _{1},\gamma _{2},\gamma _{3},a) \\ I_3= g_{3}(\gamma _{1},\gamma _{2},\gamma _{3},a) \\ I_4= g_{4}(\gamma _{1},\gamma _{2},\gamma _{3},a) \end{array}\right. } \end{aligned}$$

has a unique solution in terms of $\gamma _{1},\gamma _{2},\gamma _{3}$ and *a*:

9 $$\begin{aligned} {\left\{ \begin{array}{ll} \gamma _{1}= \gamma _{1}(I_1, I_2, I_3,I_4) \\ \gamma _{2}= \gamma _{2}(I_1, I_2, I_3,I_4) \\ \gamma _{3}= \gamma _{3}(I_1, I_2, I_3,I_4) \\ a= a(I_1, I_2, I_3,I_4) \end{array}\right. }. \end{aligned}$$

Substituting the solution (9) in (1), (2) and (5), it is possible to obtain, respectively, the expressions of the *df*, the *pdf* and *p*th quantile the in terms of the chosen indicators. So, for example, the distribution function of reparameterized Beta-SM4 is

10 $$\begin{aligned}&F_{R-Beta-SM4}(x; I_1, I_2, I_3, I_4)= \nonumber \\&\left[ 1-\left( 1+\gamma _{2}(I_1, I_2, I_3,I_4) x^{\gamma _{3}(I_1, I_2, I_3,I_4)} \right) ^{-\gamma _{1}(I_1, I_2, I_3,I_4)}\right] ^{a(I_1, I_2, I_3,I_4)}. \end{aligned}$$

Now, in order to evaluate how climatic or physic characteristics could affect the chosen indicators, we will express them as functions of a set of covariates that could have an effect separately and/or simultaneously. If for each sampled catchment *i*, $(i=1,...,n)$, an hydrological variable of interest (e.g. annual streamflow maximum) and a set of explanatory covariates are observed, indicators (8) can be reformulated by specifying their relationship with covariates. Denoting by $\varvec{w}_{1,i}$, $\varvec{w}_{2,i}$, $\varvec{w}_{3,i}$ and $\varvec{w}_{4,i}$ the vectors that, in general, affect the four indicators separately, the relationship between indicators and covariates can be specified as follow

11 $$\begin{aligned} I_{j,i}=h_j(\varvec{w}_{j,i},\varvec{\beta }_j) \end{aligned}$$

for $j=1,2,3,4$ and $i=1,...,n$, where $h(\cdot )$ is an appropriate link function chosen according to whether $I_{j,i}$ is positive or varies in (0, 1). Parameters $\varvec{\beta }_j$ indicate the regression coefficients associated with the covariates that need to be estimated on the basis of available observations by the maximum likelihood method. It is worth to note that this approach allow to take into account also the possible nonlinearity between the hydrological variable of interest and covariates since all the parameters of the conditional distribution of the response can be modelled through parametric linear or non-linear functions of explanatory variables. Moreover, we are able to obtain estimation of measures of interest, such as flood quantiles, also for ungauged catchments or for catchments having short data records.

### Formulation in terms of median and return level

In the following subsection we propose a particular reparameterization of Beta-SM4 distribution, involving median and return level as indicators of interest. Both these indicators have a simple and direct interpretation in terms of hydrologic meaning and the inspection of the possible effect of some covariates on them could be of particular interest in many real contexts. This particular reparameterization is just a possible example, indeed different reformulations could be considered, depending on the features to be investigated.

The original parameters are substituted by the following one-to-one transformation

12 $$\begin{aligned} (\gamma _1, \gamma _2, \gamma _3,a) \mapsto (\tau , me, x_0, a) \end{aligned}$$

where $\tau =\frac{1}{\gamma _1}$, *me* is the median of distribution, $x_0$ is the return level, corresponding to a pre-fixed return period $\pi _{x_0}$, and parameter *a* remain unchanged.

The adopted reparameterization for $\gamma _1$ is similar to the one proposed in^69^ for Dagum distribution and it is especially convenient when the feature of interest is transferable, since it is a direct indicator of concentration level. For the sake of generality, we mantain this reparemeterization as it could be usefull in other contexts of study.

From (6) and (7), after simple algebra, we obtain

13 $$\begin{aligned} {\left\{ \begin{array}{ll} \gamma _{1}= \frac{1}{\tau } \\ \gamma _{2}= [(1-0.5^{1/a})^{-\tau }-1] \cdot me^{-\frac{\log \left\{ \left[ 1-\left( 1-\frac{1}{\pi _{x_0}}\right) ^{1/a}\right] ^{-\tau }-1\right\} - \log \left\{ [1-0.5^{1/a}]^{-\tau }-1\right\} }{\log x_0- \log me}} \\ \gamma _{3}=\frac{\log \left\{ \left[ 1-\left( 1-\frac{1}{\pi _{x_0}}\right) ^{1/a}\right] ^{-\tau }-1\right\} - \log \left\{ [1-0.5^{1/a}]^{-\tau }-1\right\} }{\log x_0- \log me}\\ a= a \end{array}\right. }. \end{aligned}$$

From (10) and (13), it is immediate to obtain the new expression of *df* of Beta-SM4 *r.v.* in terms of median and return level:

14 $$\begin{aligned} F_{R1-Beta-SM4}(x;\tau ,me,x_0,a)= \left[ 1-\left( 1+\left[ \left( 1-0.5^{\frac{1}{a}}\right) ^{-\tau }-1\right] \right. \right. \nonumber \\ \left. \left. \left( \frac{x}{me}\right) ^{\frac{\log \left\{ \left[ 1-\left( 1-\frac{1}{\pi _{x_0}}\right) ^{1/a}\right] ^{-\tau }-1\right\} - \log \left\{ [1-0.5^{1/a}]^{-\tau }-1\right\} }{\log x_0- \log me}} \right) ^{-\frac{1}{\tau }}\right] ^{a} \end{aligned}$$

In order to identify a specific link function, we observe that all the indicators involved in the reparameterization (ie $\tau$, *me*, $x_0$ and *a*) are positive; in this context, it is usual to choose a log-linear link, ie $\exp ({\textbf {w}}'\varvec{\beta })$, where ${\textbf {w}}'\varvec{\beta }$ is the linear predictor.

## Estimation

By specifying relationship between indicators and covariates as in (11), the general parameterization in Eq. (9) may be rewritten as

15 $$\begin{aligned} {\left\{ \begin{array}{ll} \breve{\gamma }_{1,i}= \gamma _{1}(I_{1,i}, I_{2,i}, I_{3,i},I_{4,i}) \\ \breve{\gamma }_{2,i}= \gamma _{2}(I_{1,i}, I_{2,i}, I_{3,i},I_{4,i}) \\ \breve{\gamma }_{3,i}= \gamma _{3}(I_{1,i}, I_{2,i}, I_{3,i},I_{4,i}) \\ \breve{a}_{i}= a(I_{1,i}, I_{2,i}, I_{3,i},I_{4,i}) \end{array}\right. }. \end{aligned}$$

Consequently the log-likelihood function expressed in terms of the unknow coefficients $\varvec{\beta }=(\varvec{\beta '}_{1},\varvec{\beta '}_{2},\varvec{\beta '}_{3},\varvec{\beta '}_{4})'$ is given by

16 $$\begin{aligned} \ell (\varvec{\beta };\varvec{x},\varvec{W})=\sum _{i=1}^n \ln {\breve{a}_i}+\sum _{i=1}^n (\breve{a}_i-1) \ln [F_{SM}(x_i;\breve{\varvec{\gamma }}_i)]+\sum _{i=1}^n\ln [f_{SM}(x_i;\breve{\varvec{\gamma }}_i)] \end{aligned}$$

where $\varvec{W}$ denotes the matrix containing the covariates for the four indicators and $\varvec{\breve{\gamma }'}_{i}=(\breve{\gamma }_1,\breve{\gamma }_2,\breve{\gamma }_3)$. Assuming that the vectors of regression coefficients are of size $p_j$ for $j=1,2,3,4$, the $(p_1+p_2+p_3+p_4)$ likelihood equations are

17 $$\begin{aligned} \frac{\partial \ell (\varvec{\beta };\varvec{x},\varvec{W})}{\partial {\beta _{j,r_j}}}&=\sum _{i=1}^n \frac{\dot{a}_{j,r_j,i}}{\breve{a}_i}\nonumber \\&\quad + \sum _{i=1}^n \biggl [\dot{a}_{j,r_j,i} \ln [F_{SM}(x_i;\breve{\varvec{\gamma }}_i)]+(\breve{a}_i-1) \frac{\dot{F}_{SM}(x_i;\breve{\varvec{\gamma }}_i)_{j,r_j,i}}{F_{SM}(x_i;\breve{\varvec{\gamma }}_i)} \biggl ] \nonumber \\&\quad + \sum _{i=1}^n \frac{\dot{f}_{SM}(x_i;\breve{\varvec{\gamma }}_i)_{j,r_j,i}}{f_{SM}(x_i;\breve{\varvec{\gamma }}_i)}=0 \end{aligned}$$

where the quantities $\dot{a}_{j,r_j,i}$, $\dot{F}_{SM}(x_i;\breve{\varvec{\gamma }}_i)_{j,r_j,i}$ and $\dot{f}_{SM}(x_i;\breve{\varvec{\gamma }}_i)_{j,r_j,i}$ denote the partial derivatives of $\breve{a}_i$, $F_{SM}(x_i;\breve{\varvec{\gamma }}_i)$ and $f_{SM}(x_i;\breve{\varvec{\gamma }}_i)$ with respect to the parameter $\beta _{j,r_j}$, for $j=1,2,3,4$ and $r_j=1,2,...,p_j$, *i.e.*

$$\begin{aligned} \dot{a}_{j,r_j,i}=\frac{\partial \breve{a}_i}{\partial \beta _{j,r_j}}=\frac{\partial \breve{a}_i}{\partial I_{j, i}}\times \frac{\partial I_{j, i}}{\partial \beta _{j,r_j}}, \end{aligned}$$

$$\begin{aligned} \dot{F}_{SM}(x_i;\breve{\varvec{\gamma }}_i)_{j,r_j,i}= & {} \frac{\partial F_{SM}(x_i; I_{1,i}, I_{2,i}, I_{3,i}, I_{4,i}) }{\partial \beta _{j,r_j}} \\= & {} \frac{\partial F_{SM}(x_i; I_{1,i}, I_{2,i}, I_{3,i}, I_{4,i}) }{\partial I_{j,i}} \times \frac{ \partial I_{j, i}}{\partial \beta _{j,r_j}} \end{aligned}$$

and

$$\begin{aligned} \dot{f}_{SM}(x_i;\breve{\varvec{\gamma }}_i)_{j,r_j,i}= & {} \frac{\partial f_{SM}(x_i; I_{1,i}, I_{2,i}, I_{3,i}, I_{4,i}) }{\partial \beta _{j,r_j}} \\= & {} \frac{\partial f_{SM}(x_i; I_{1,i}, I_{2,i}, I_{3,i}, I_{4,i}) }{\partial I_{j,i}} \times \frac{ \partial I_{j, i}}{\partial \beta _{j,r_j}}. \end{aligned}$$

The coefficients $\varvec{\beta }_j$ will highlight the impact of the corresponding covariates directly on indicators $I_{j,i}$ of interest, $j=1,2,3,4$.

The system of the likelihood equations in (17) does not admit any explicit solution therefore, the *ML* estimates $\hat{\beta }_{j,r_j}$, for $j=1,2,3,4$ and $r_j=1,2,...,p_j$, can only be obtained by means of numerical procedures. Under the usual regularity conditions, the known asymptotic properties of the maximum likelihood method ensure that $\sqrt{n}(\hat{\varvec{\beta }}_n-\varvec{\beta })\xrightarrow {d}N(\varvec{0},\varvec{\Sigma }_{\varvec{\beta }})$, where $\varvec{\Sigma }_{\varvec{\beta }}=[\lim _{n\rightarrow \infty }\varvec{I(\varvec{\beta })}/n]^{-1}$ is the $(p_1+p_2+p_3+p_4)\times (p_1+p_2+p_3+p_4)$ asymptotic variance-covariance matrix and $\varvec{I(\varvec{\beta })}$ is the Fisher information matrix, given by $\varvec{I(\varvec{\beta })}=-E\left( {\textbf {H}}\right)$ where ${\textbf {H}}$ is the Hessian matrix of the second partial derivatives of the log-likelihood function, *ie* $\frac{\partial ^2 \ell (\varvec{\beta };\varvec{x},\varvec{W})}{\partial {\beta _{j,r_j} \beta _{h,r_h }}}$. The elements of the Fisher information matrix can be determined in a similar way to what was done in^69^ and are available on request.

## Application

**Figure 1:**
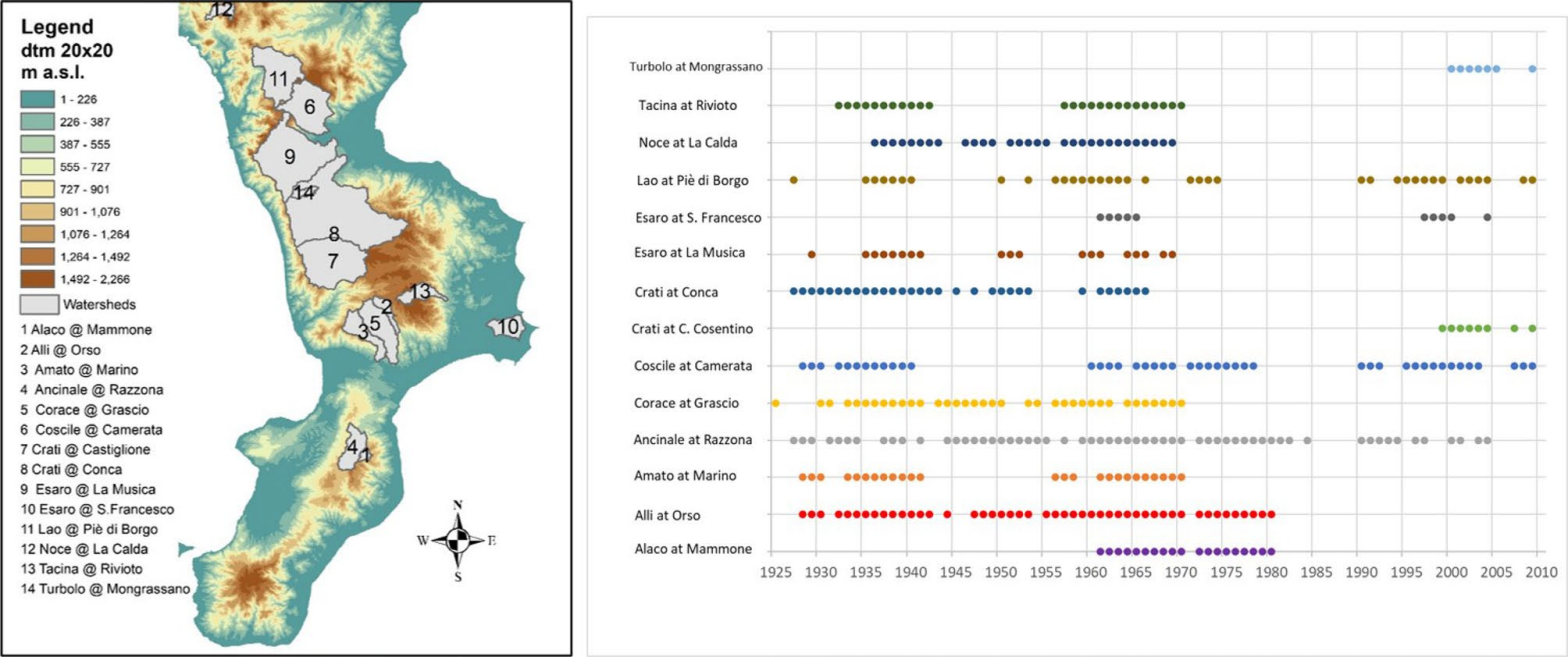
Watersheds location, obtained using ArcGIS Desktop: Release 10.3.1. Redlands, CA: Environmental Systems Research Institute (left panel) and extent of the observation period for each recording gauge (right panel).

In this example, we consider real time series of annual streamflow maxima data relative to a set of 14 catchments located in Calabria or partially included in the Basilicata region, southern Italy (Fig. 1, left panel). Timeseries for the 14 stream gauges refer to the official and validated database of the “Centro Funzionale Multirischi” of the Calabria Region (data are available upon request at https://www.cfd.calabria.it/) that is the institution in charge for data collection and management. Data are annual maxima obtained from hourly or sub-hourly resolution discharge measurements. The series have different length, ranging from 7 to 59 observations and cover non-homogeneous periods, from 1925 to 2009 (Fig. 1, right panel). Selected catchments range in size from 27 to 1323 km^2^, while mean elevation varies from about 300 m to more than 1300 m a.s.l.. Table 1 summarizes some geomorphoclimatic characteristics of the investigated catchments. The area is characterized by a Mediterranean climate, with rainy periods mainly coinciding with fall and winter months while summers are hot and dry, strongly affecting the seasonal runoff cycle of the streams. Nevertheless, there are considerable differences in temperature and rainfall heights between mountainous territory in interior areas and coastal zones. The mean annual precipitation over the catchment set averages approximately 1000 mm: it is usually greater than 1100 mm for elevations above of 500 m a.s.l. and decreases to 700 mm in the Ionian coast (east coast).

**Table 1:** Geomorphoclimatic characteristics of the investigated catchments: A is the catchment area, *n* is the record length in years; Hm is the mean catchment elevation; Ybar is the latitude of the center of the basin; LCV6 is the LCV of annual maxima of rainfall heights for a duration of six hours; ${\hbox {Q}}_{\hbox {m}}$ is the mean value of the annual streamflow maxima; S.D. is the standard deviation of annual maxima time series.

#	Catchment	*n*	A km^2^	Hm (m a.s.l.)	Ybar (km)	LCV6 (-)	${\hbox {Q}}_{\hbox {m}}$ (m^3^/s)	S.D. (m^3^/s)
1	Alaco at Mammone	19	16.16	1045.22	4.272.031	0.255	13.6	10.2
2	Alli at Orso	47	46.47	1143.60	4.328.678	0.205	16.7	11.9
3	Amato at Marino	25	111.67	758.84	4.319.942	0.217	68.8	78.62
4	Ancinale at Razzona	59	112.06	818.54	4.274.622	0.258	82.9	62.95
5	Corace at Grascio	36	177.34	821.98	4.321.842	0.218	154.8	107.44
6	Coscile at Camerata	44	274.5	748.91	4.408.370	0.185	77.2	51.98
7	Crati at Castiglione C.	8	403.61	716.85	4.347.844	0.188	343.9	172.27
8	Crati at Conca	31	1323.71	664.5	4.361.380	0.194	441.4	230.92
9	Esaro at La Musica	19	537.37	520.18	4.388.924	0.219	328.8	268.06
10	Esaro at S. Francesco	10	87.89	111.46	4.322.285	0.276	389.0	175.68
11	Lao at Piè di Borgo	37	281.86	846.67	4.421.835	0.163	165.2	114.31
12	Noce at La Calda	30	41.59	1074.06	4.446.148	0.177	30.7	12.67
13	Tacina at Rivioto	25	77.07	1302.87	4.335.194	0.243	81.2	103.16
14	Turbolo at Mongrassano	7	27.93	306.92	4.374.993	0.183	27	11.69

**Figure 2:**
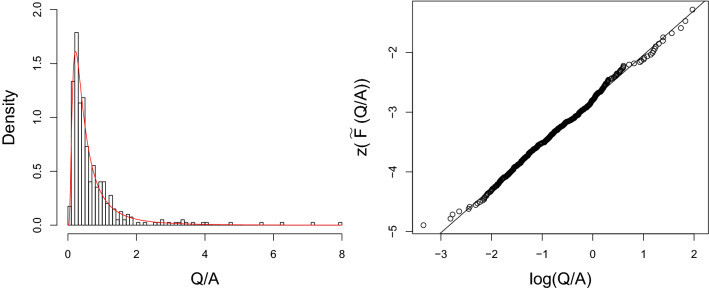
Empirical and fitted density curve (left panel) and probability plot (right panel), under the homogeneity hypothesis.

In order to show the adequacy of the proposed model in describing this kind of data, we consider the reparameterization reported in (12), involving the four indicators $I_1=\tau$, $I_2=me$, $I_3=x_0$ and $I_4=a$, where $I_1$ is a direct indicator of distribution concentration, $I_2$ is the median of streamflow maxima, $I_3$ is chosen to be the 5-years return level and $I_4$ is equal to the parameter *a* in the original parameterization. First of all, we obtain the maximum likelihood estimates (MLEs) of the parameters under the homogeneity hypothesis of the catchments, i.e. the estimates obtained in absence of covariates effects. To this end, we consider the udometric coefficient, to take into account the different basin areas. The obtained MLEs and corresponding $95\%$ confidence interval (in brackets) are: $\hat{\tau }=1.87\times 10^{-5}$ $(1.59\times 10^{-5}; 2.20\times 10^{-5})$, $\hat{me}=0.449$ (0.393; 0.513), $\hat{x_0}=0.958$ (0.806; 1.139) and $\hat{a}=129.13$ (112.8; 147.8). The adequacy of the model to the analyzed data is graphically confirmed from the probability plot presented in Fig. 2: the trend appears to be linear by fitting a straight line through the points, suggesting that the Beta-SM4 is an appropriate model for these data. The details of the construction of the probability plot are given in the *Appendix* (see Supplementary Information).

Focusing on data set of udometric coefficient for Calabria, the obtained Beta-SM4 performances were compared, besides with the widely used Generalized Extreme Value (GEV) distribution, also with the Two-Component Extreme Value (TCEV) distribution^13,43,70^, that is a 4-parameter probability function and it is widely adopted for RFFA in Calabria region. The underlying hypothesis for the TCEV formulation is the existence of two kinds of flood populations for series of annual maximum flows of many Italian rivers, and particularly in the Mediterranean area. This theoretical consideration can be reconducted to different physical interpretations of the event genesis: ordinary floods are generated by frontal-type rainfalls, which is the most frequent type of rainfall and produces smaller events; conversely, extraordinary floods are less frequent, more severe and mostly generated by heavy convective rainfall events. At this step, authors considered MLEs for the parameters under the homogeneity hypothesis of the catchments for GEV and TCEV. Moreover, as a global selection criterion, Akaike Information Criterion (AIC^71^), is evaluated. The result obtained for Beta-SM4 ($AIC=414,613$) suggests a better and a similar performance, when compared, respectively, with that for TCEV ($AIC=438,787$) and GEV ($AIC=413,667$). In order to better interpretate the obtained AIC values and to better quantify the information loss experienced by using Beta-SM4 or TCEV rather than GEV, it is convenient to rescale AIC values to the differences between each AIC value and their minimum (see e.g.^72^, pp. 270–271 and^73^, section 2.6). By considering the frequently adopted rule of thumb for assessing the relative merits of a model, it can be concluded that the Beta-SM4 represents a good alternative to the GEV distribution (difference equal to 0.946 and therefore lower than 2), while the TCEV distribution have essentially no support (difference equal to 25.12 and therefore substantially greater than 10).

**Figure 3:**
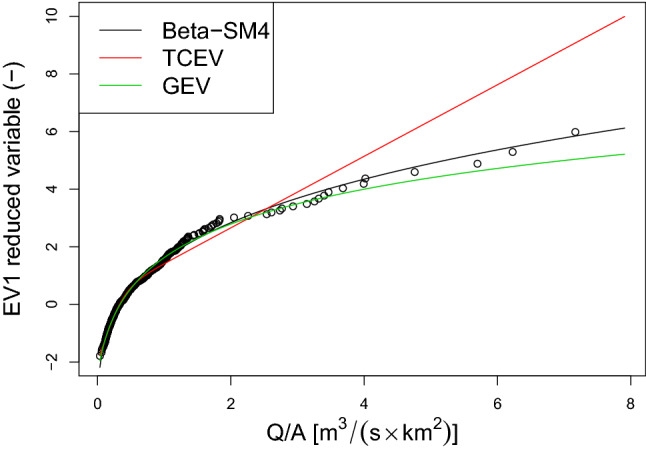
Comparison between Beta-SM4 and TCEV distributions for udometric coefficient data set of Calabria.

**Figure 4:**
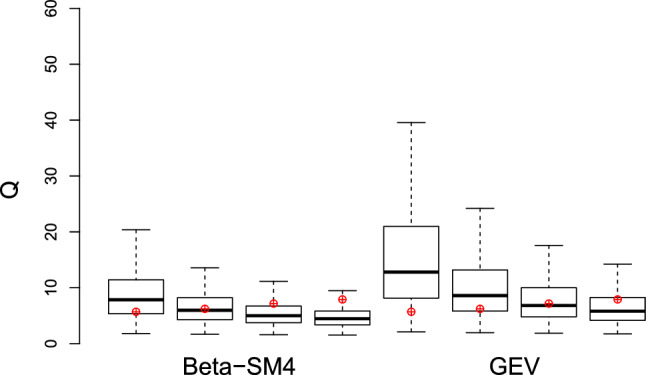
Box-plot of quantiles obtained from resampling procedure, sorted in decreasing order of *i*. Observed quantiles of udometric coefficient are depicted in red.

This conclusion is supported by the graphical comparison reported in an EV1 probabilistic plot (Fig. 3), from which it is clear that Beta-SM4 model performs better than TCEV and it is a valid alternative to the GEV model, especially in describing the extreme right tail. To explore more in depth the performance of these two models in describing extreme quantiles, we consider the resampling procedure reported in^74^ and already recalled in^23^. In this case, we follow the procedure fitting models on 1000 bootstrap samples of size 50 and extrapolating the $i-th$ extreme right-tail quantiles, corresponding to the empirical cumulative probability given by $\frac{i}{N+1}$ (where $N=397$ is the total number of observation and $i=394,395,396,397$). Figure 4 shows the sample characteristics of these extreme quantiles and the corresponding observed quantiles, depicted in red. The simulation shows that GEV model tends to overrepresent the extreme quantiles, while Beta-SM4 shows a better performance, confirming the findings already reported in^23^ on different data. These preliminary evidences suggest that a regression procedure based on Beta-SM4 could be suitable for investigating the impact of some covariates on streamflow maxima distributions and its features. In particular, the reparameterization proposed in “The Model” section will allow to explore the possible effects on the median and 5-years return level for each catchment. To this end, we consider some catchments characteristics, such as the latitude of the centre of the basin (Ybar, in tens of km), the catchment area (A, in km^2^), the mean elevation (Hm, m.a.s.l.) and the sample values of the coefficient of L-variation of annual maxima of rainfall heigths with a duration of six hours (LCV6). We consider the reparameterization reported in (12) and since the indicators are all positive the most appropriate link function appears to be that the log-linear to relate the above mentioned characteristics to the indicators, ie $\tau =\exp (\varvec{w'}_{1} \cdot \varvec{\beta }_1)$, $me=\exp (\varvec{w'}_{2} \cdot \varvec{\beta }_2)$, $x_0=\exp (\varvec{w'}_{3} \cdot \varvec{\beta }_3)$ and $a=\exp (\varvec{w'}_{4} \cdot \varvec{\beta }_4)$.

Table 2 reports the obtained MLEs of the parameters, the corresponding standard errors (SE) and the results from Wald test (*t* and *p* value) for statistical significance of parameters related to the four indicators $I_j$ for $j=1,...4$. As we expected, the considered variables seem to have a significant influence on the median of annual streamflow maxima distribution and on return level. In particular both median and return level seem to be positively associated with Ybar, LCV6 and A, while streamflow decreases as the mean elevation Hm of basin increases. Having obtained the MLEs, it is also possible to look at the fitted distributions for each catchment $F_{Beta-SM4-I}(x;\hat{\tau }_i,\hat{me}_i,\hat{x}_{0_i},\hat{a}_i)$, for $i=1,...,14$, where the generic indicator is obtained substituting in (11) the estimates of regression coefficients and covariates values for the catchment. To obtain the MLE estimates we considered numerical procedure based on a quasi-Newton method (BFGS method implemented in R), imposing some constraints to ensure admissible results.

**Figure 5:**
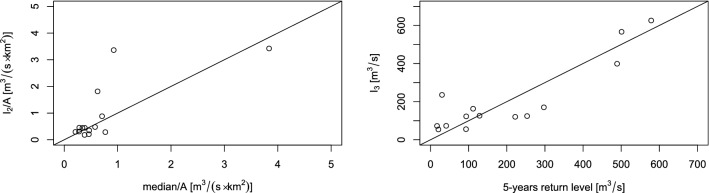
Empirical medians and return levels versus relative fitted indicators.

Observed versus estimated median and 5-years return level for different catchments is compared in Fig. 5. In this case, median values were rescaled by considering the catchment area. As it can be seen, fitted and observed values are similar, except for some catchment, confirming that this regression approach allows to adequately and simultaneously estimate substantial features of streamflow maxima in presence of heterogeneity.

**Table 2:** MLEs of the parameters (log-likelihood: − 2146.313).

Covariate	Estimate (95% CI)	SE	t	*p* value
$$\tau =\exp (\varvec{w'}_{1} \cdot \varvec{\beta }_1)$$				
Intercept	$-10.865$ $(-11.016; -10.715)$	0.077	$-141.18$	$<0.001$
$$me=\exp (\varvec{w'}_{2} \cdot \varvec{\beta }_2)$$				
Intercept	$-25.435$ $(-26.017;-24.853)$	0.297	$-85.620$	$<0.001$
Ybar	0.065 (0.064; 0.066)	$6.42 \times 10^{-4}$	100.99	$<0.001$
LCV6	11.464 (10.843; 12.086)	0.317	36.131	$<0.001$
A	0.0016 (0.0014; 0.0018)	$9.44\times 10^{-5}$	16.834	$<0.001$
Hm	$-0.0018$ $(-0.0021; -0.0014)$	$1.77\times 10^{-4}$	$-9.981$	$<0.001$
$$x_0=\exp (\varvec{w'}_{3} \cdot \varvec{\beta }_3)$$				
Intercept	$-9.037$ $(-9.094; -8.979)$	0.029	$-309.61$	$<0.001$
Ybar	0.032 (0.030; 0.033)	$5.28\times 10^{-4}$	59.65	$<0.001$
LCV6	6.684 (5.922; 7.446)	0.389	17.19	$<0.001$
A	0.0012 (0.0010; 0.0015)	$1.40\times 10^{-4}$	8.768	$<0.001$
Hm	$-0.0018$ $(-0.0023;-0.0013)$	$2.38\times 10^{-4}$	$-7.502$	$<0.001$
$$a=\exp (\varvec{w'}_{4} \cdot \varvec{\beta }_4)$$				
Intercept	5.849 (5.297; 6.401)	0.282	20.769	$<0.001$

**Figure 6:**
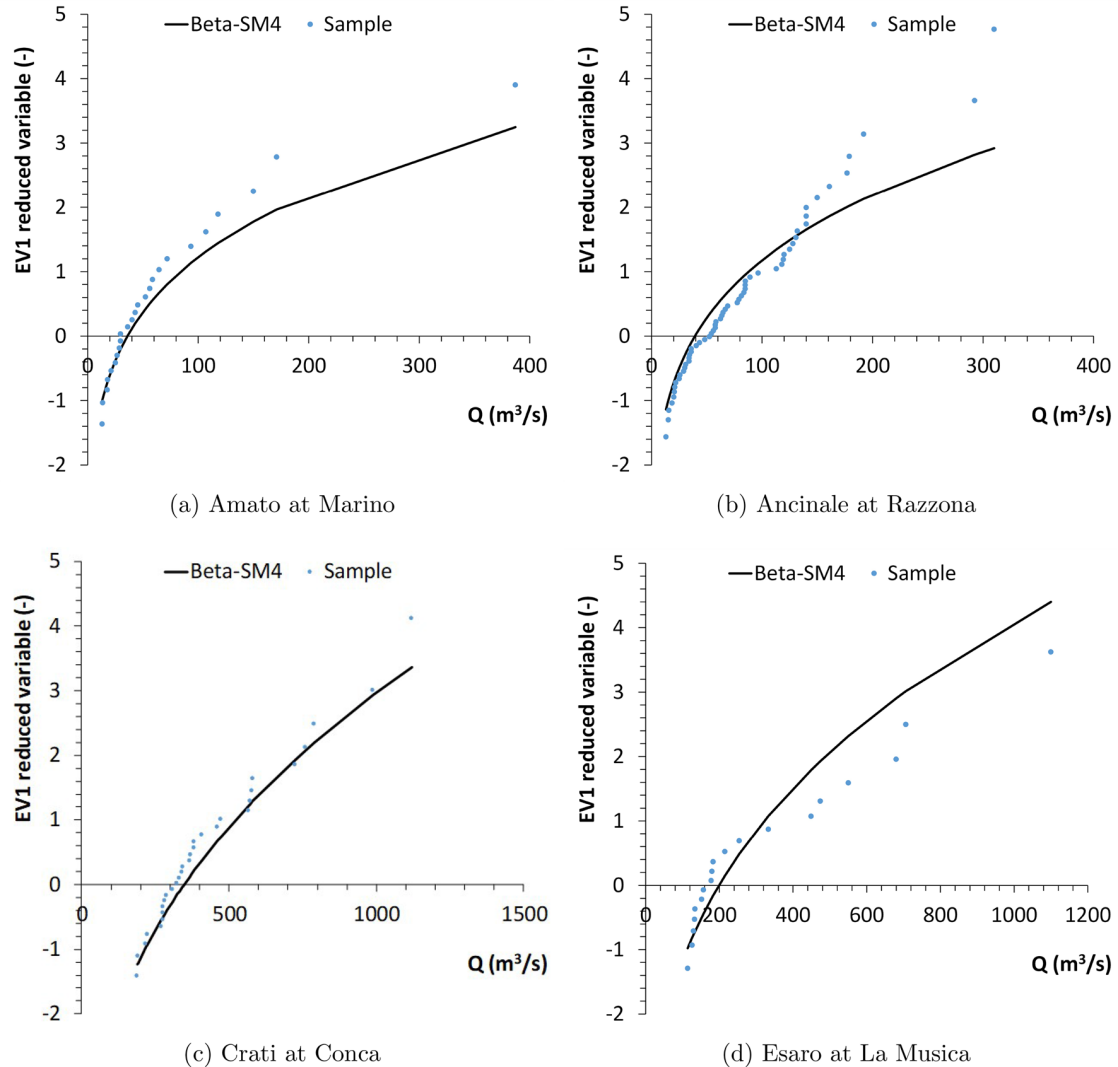
EV1 probabilistic plot of Beta-SM4 for some cathments.

It is worth to note that the dependence find in the regressive structures is of general nature and not ensure a causal relationship between floods and covariates^75^. The derivation of relationships intended for practical applications require interpretation from a hydrological perspective and further investigations related to the use of different descriptors, model robustness, model efficiency and associated uncertainties that are beyond the scope of this paper. Another aspect to be investigated for hydrological extremes should be the potential presence of long-range dependence or strong clustering (grouping) of similar values, or the Hurst phenomenon^76–81^, which is quite common in natural processes. However, the annual maxima series usually tend to hide the Hurst behaviour, as explained in^20,82^. In addition, for the selected case studies, this analysis cannot be easily carried out, because it would require datasets without missing data, while many “holes” are present in the investigated time series (Fig. 1, right panel). For the same reason, it clearly difficult to also evaluate the eventual existence of a compound effect, which indicates that if these catchments are close to each other, then the probability occurrence of an extreme value to one site may be overestimated if the same extreme storm event caused an extreme value in an adjacent catchment. In fact, missing data could make unreliable a multivariate analysis, as proposed in^83^. As examples of application for specific catchments, the results for Amato at Marino, Ancinale at Razzona, Crati at Conca and Esaro at La Musica are reported in Fig. 6.

## Conclusions

In this paper we proposed a new parameterization of the Beta-Singh-Maddala distribution in order to model extreme hydrologic events and simoultaneously investigate on their possible determinants. As shown in^23^, this distribution is related to the Dagum distribution, recently considered in the context of analysis of hydrologic extreme events^12^ and, owing to the fact that it can be viewed as a generalization of a Beta-p distribution, to other distributions frequently used in the specific literature. In presenting the general reparameterization it is assumed that there are four indicators, functions of the four parameters of the distribution, of specific interest in the hydrological field and a set of explanatory covariates. The particular case in which interest is placed on the median and on the return level is also presented. Finally, an application to a real hydrologic dataset is reported. The application results confirm that the proposed parameterization well describes the observed data and allows for an understanding on the effects of covariates on interest indicators, such as median and return level. The obtained findings suggest that the proposed reparameterization of Beta-Singh-Maddala distribution can be considered as a valid alternative to some classical models for extreme value analysis, simoultaneously allowing for a direct interpretation in terms of particular factors impact on aspects of hydrological interest.

## Supplementary Information

Supplementary Information.

## References

[1] Katz R, Brush G, Parlange M (2005). Statistics of extremes: Modeling ecological disturbances. Ecology.

[2] Tebaldi C, Hayhoe K, Arblaster J, Meehl G (2006). Going to the extremes. Clim. Change.

[3] Bobee B (1975). The log Pearson type 3 distribution and its application in hydrology. Water Resour. Res..

[4] Johnson NL, Kotz S, Balakrishnan N (1994). Continuous Univariate Distributions.

[5] Hosking JRM, Wallis JR (1987). Parameter and quantile estimation for the generalized Pareto distribution. Technometrics.

[6] Dargahi-Noubary GR (1989). On tail estimation: An improved method. Math. Geol..

[7] Balakrishnan N, Leung MY (1988). Means, variances and covariances of order statistics, BLUEs for the Type-I generalized logistic distribution, and some applications. Commun. Stat. Simul. Comput..

[8] Dyrrdal, A. V. Estimation of extreme precipitation in Norway and a summary of the state of the art. Report No. 08/2012, Climate, Norwegian Meteorological Institute (2012).

[9] Bücher A, Lilienthal J, Kinsvater P, Fried R (2021). Penalized quasi-maximum likelihood estimation for extreme value models with application to flood frequency analysis. Extremes.

[10] Mujere N (2011). Flood frequency analysis using the Gumbel distribution. Int. J. Comput. Sci. Eng..

[11] Jeong BY, Murshed SM, Seo YA, Park JS (2014). A three-parameter kappa distribution with hydrological application: A generalized Gumbel distribution. Stoch. Environ. Res. Risk Assess..

[12] Murshed SM, Kim S, Park JS (2011). Beta-k distribution and its application to hydrologic events. Stoch. Env. Res. Risk Assess..

[13] Rossi F, Fiorentino M, Versace P (1984). Two-component extreme value distribution for flood frequency analysis. Water Resour. Res..

[14] Zakerzadeha H, Dolati A (2009). Generalized Lindley distribution. J. Math. Ext..

[15] Domma F, Condino F (2013). The Beta-Dagum distribution: Definition and properties. Commun. Stat. Theory Methods.

[16] Hussain T, Bakouch HS, Iqbal Z (2017). A new probability model for hydrologic events: Properties and applications. Jo. Agricol. Biol. Environ. Stat..

[17] Paranìba PF, Ortega EMM, Cordeiro GM, Pescima RR (2011). The beta Burr XII distribution with application to lifetime data. Comput. Stat. Data Anal..

[18] Icobellis V, Fiorentino M (2000). Derived distribution of floods based on the concept of partial area coverage with a climatic appeal. Water Resour. Res..

[19] De Michele CA, Salvadori G (2002). On the derived flood frequency distribution: Analytical formulation and the influence of antecedent soil moisture condition. J. Hydrol..

[20] Koutsoyiannis D (2021). Stochastics of Hydroclimatic Extremes—A Cool Look at Risk.

[21] Marani M, Ignaccolo M (2015). A metastatistical approach to rainfall extremes. Adv. Water Resour..

[22] Zorzetto E, Botter G, Marani M (2016). On the emergence of rainfall extremes from ordinary events. Geophys. Res. Lett..

[23] Domma F, Condino F (2016). Use of the Beta-Dagum and Beta-Singh-Maddala distributions for modeling hydrologic data. Stoch. Environ. Res. Risk Assess..

[24] Chebana, F. & Ouarda, T. B. M. J. Depth and homogeneity in regional flood frequency analysis. *Water Resour. Res.* **44** (11) (2008).

[25] Gruber, A. M. & Stedinger, J. R. Models of LP3 regional skew, data selection, and Bayesian GLS regression. In *World Environmental & Water Resources Conference* 12–16 (2008).

[26] Micevski, T. & Kuczera, G. Combining site and regional flood information using a Bayesian Monte Carlo approach. *Water Resour. Res.* **45** (2009).

[27] Gaume E, Gaál L, Viglione A, Szolgay J, Kohnová S, Blöschl G (2010). Bayesian MCMC approach to regional flood frequency analyses involving extraordinary flood events at ungauged sites. J. Hydrol..

[28] Martel B, Ouarda T, Barbet M, Bruneau P, Latraverse M, Nezhad MK (2011). Regional frequency analysis of autumnal floods in the province of Quebec. Canada. Nat. Hazards.

[29] Nezhad MK, Chokmani K, Ouarda T, Barbet M, Bruneau P (2010). Regional flood frequency analysis using residual kriging in physiographical space. Hydrol. Process..

[30] Nyeko-Ogiramoi P, Willems P, Mutua F, Moges SA (2012). An elusive search for regional flood frequency estimates in the River Nile basin. Hydrol. Earth Syst. Sci..

[31] Ahn K, Palmer R (2016). Regional flood frequency analysis using spatial proximity and basin characteristics: Quantile regression vs. parameter regression technique. J. Hydrol..

[32] Ojha R, Tripathi S (2018). Using attributes of ungauged basins to improve regional regression equations for flood estimation: A deep learning approach. ISH J. Hydraul. Eng..

[33] Wallis JR, Matalas NC, Slack JR (1974). Just a moment!. Water Resour. Res..

[34] Dalrymple T. Flood frequency analysis. U.S. geological survey. *Water Supply Paper* 1543-A (1960).

[35] Smith A, Sampson C, Bates P (2015). Regional flood frequency analysis at the global scale. Water Resour. Res..

[36] Hosking JRM, Wallis JR (1997). Some statistics useful in regional frequency analysis. Water Resour. Res..

[37] Abida H, Ellouze M (2008). Probability distribution of flood flows in Tunisia. Hydrol. Earth Syst. Sci..

[38] Hussain T, Pasha G (2009). Regional flood frequency analysis of the seven sites of Punjab, Pakistan, using L-moments. Water Resour. Manag..

[39] Noto VL, La Loggia G (2009). Use of L-moments approach for regional flood frequency analysis in Sicily, Italy. Water Resour. Manag..

[40] Saf B (2009). Regional flood frequency analysis using L- moments for the West Mediterranean region of Turkey. Water Resour. Manag..

[41] Seckin N, Haktanir T, Yurtal R (2011). Flood frequency analysis of Turkey using L-moments method. Hydrol. Process..

[42] Laio F, Ganora D, Claps P, Galeati G (2011). Spatially smooth regional estimation of the flood frequency curve (with uncertainty). J. Hydrol..

[43] Biondi, D., Claps, P., Cruscomagno, F., De Luca, D. L., Fiorentino, M., Ganora, D., Gioia, A., Iacobellis, V., Laio, F., Manfreda, S. & Versace, P. After the VAPI Project: Evaluation of the design maximum floods concerning the Calabria POR project (in Italian). In *Proceedings of XXXIII Italian National Conference on Hydraulics and Hydraulic Engineering* 10–15 September 2012, Brescia, Italy (2012).

[44] Haddad K, Rahman A (2012). Regional flood frequency analysis in eastern Australia: Bayesian GLS regression-based methods within fixed region and ROI framework—quantile regression vs. parameters regression technique. J. Hydrol..

[45] Aydoğan D, Kankal M, Onsoy H (2016). Regional flood frequency analysis for Çoruh Basin of Turkey with L-moments approach. J. Flood Risk Manag..

[46] Ouarda TBMJ (2013). Hydrological frequency analysis, regional. Encycl. Environ..

[47] Farsadnia F, Kamrood MR, Nia AM, Modarres R, Bray MT, Han D, Sadatinejad J (2014). Identification of homogeneous regions for regionalization of watersheds by two-level self-organizing feature maps. J. Hydrol..

[48] Burn DH, Zrinji Z, Kovvalchuck M (1997). Regionalisation of catchments for regional flood frequency analysis. J. Gydrol. Eng..

[49] Chebana F, Ouarda TBMJ (2007). Mulivariate L-moment homogeneity test. Water Resour. Res..

[50] Ouarda TBMJ, Cunderlik JM, St-Hilaire A, Barbet M, Bruneau P, Bobée B (2006). Data-based comparison of seasonality-based regional flood frequency methods. J. Hidrol..

[51] Ouarda TBMJ, St-Hilaire A, Bobée B (2008). Synthgése des dévelopments récents en analyse régionale des extremes hydrologiques/A review of recent developments in regional frequency analysis of hydrological extremes. Revue des Sciences de l’eau/J Watr Sci.

[52] Ouarda TBMJ (2008). Intercomparison of regional flood frequency estimation methods at ungauged sites for a Mexican case study. J. Hydrol..

[53] Haddad K, Rahman A, Zaman M, Shrestha S (2013). Applicability of Monte Carlo cross validation technique for model development and validation using generalised least squares regression. J. Hydrol..

[54] Goyal MK, Gupta V (2014). Identification of homogeneous rainfall regimes in Northeast Region of India using fuzzy cluster analysis. Water Resour. Manag..

[55] Saunders KR, Stephenson AG, Karoly DJ (2021). A regionalisation approach for rainfall based on extremal dependence. Extremes.

[56] Cassalho F, Beskow S, de Mello CR, de Moura MM, de Oliveira LF, de Aguiar MS (2019). Artificial intelligence for identifying hydrologically homogeneous regions: A state-of-the-art regional flood frequency analysis. Hydrol. Process..

[57] Rao AR, Srinivas VV, Rao AR, Srinivas VV (2008). Introduction. Regionalization of Watersheds: An Approach Based on Cluster Analysis.

[58] Beskow S, Mello CR, Vargas MM, Corrêa LL, Caldeira TL, Durães MF, Aguiar MS (2016). Artificial intelligence techniques coupled with seasonality measures for hydrological regionalization of Q90 under Brazilian conditions. J. Hydrol..

[59] Viglione A, Laio F, Claps P (2007). A comparison of homogeneity tests for regional frequency analysis. Water Resour. Res..

[60] Stedinger JR, Tasker GD (1985). Regional hydrologic analysis: 1. Ordinary, weighted, and generalized least squares compared. Water Resour. Res..

[61] Griffis VW, Stedinger JR (2007). The use of GLS regression in regional hydrologic analyses. J. Hydrol..

[62] Chokmani, K. & Ouarda, T. Physiographical space-based kriging for regional flood frequency estimation at ungauged sites. *Water Resour. Res.* **40** (2004).

[63] Skoien J, Merz R, Bloschl G (2006). Top-kriging—geostatistics on stream networks. Hydrol. Earth Syst. Sci..

[64] Rahman A, Charron C, Ouarda TBMJ, Chebana F (2018). Development of regional flood frequency analysis techniques using generalized additive models for Australia. Stoch. Env. Res. Risk Assess..

[65] Lee, T. & Ouarda, T. B. Long–term prediction of precipitation and hydrologic extremes with nonstationary oscillation processes. *J. Geophys. Res. Atmos.* **115**(D13) (2010).

[66] Marra, F., Armon, M., Adam, O., Zoccatelli, D., Gazal, O., Garfinkel, C. I., Rostkier–Edelstein, D., Dayan, U., Enzel, Y. & Morin, E. Toward narrowing uncertainty in future projections of local extreme precipitation. *Geophys. Res. Lett.* **48**(5) (2021).

[67] Ouarda TB, Yousef LA, Charron C (2019). Non-stationary intensity-duration-frequency curves integrating information concerning teleconnections and climate change. Int. J. Climatol..

[68] Singh SK, Maddala G (1976). A function for the size distribution and incomes. Econometrica.

[69] Domma F, Condino F, Giordano S (2018). A new formulation of the Dagum distribution in terms of income inequality and poverty measures. Phys. A.

[70] Versace P, Ferrari E, Gabriele S, Rossi F (1989). Valutazione delle Piene in Calabria.

[71] Akaike, H. Information theory and an extension of the maximum likelihood principle. In *Proceedings of the 2nd International Symposium on Information Theory* (eds. Petrov, B. N. & Csaki,F.) 267–281 ( Akademiai Kiado, Budapest, 1973).

[72] Burnham KP, Anderson DR (2002). Model Selection and Multimodel Inference: A Practical Information-Theoretic Approach.

[73] Burnham KP, Anderson DR (2004). Multimodel inference: Understanding AIC and BIC in model selection. Sociol. Methods Res..

[74] Wilks DS (1993). Comparison of three-parameter probability distributions for representing annual extreme and partial duration precipitation series. Water Resour. Res..

[75] Rosbjerg D, Blöschl G (2013). Prediction of floods in ungauged basins. Runoff Prediction in Ungauged Basins, Synthesis across Processes Places and Scales.

[76] Hurst HE (1951). Long-term storage capacity of reservoirs. Trans. Am. Soc. Civ. Eng..

[77] Beran J, Feng Y, Ghosh S, Kulik R (2013). Long-Memory Processes.

[78] Dimitriadis P, Koutsoyiannis D, Iliopoulou T, Papanicolaou P (2021). A global-scale investigation of stochastic similarities in marginal distribution and dependence structure of key hydrological-cycle processes. Hydrology.

[79] Klemeš V (1974). The Hurst phenomenon: A puzzle?. Water Resour. Res..

[80] Mandelbrot BB, Wallis JR (1968). Noah, Joseph, and operational hydrology. Water Resour. Res..

[81] Koutsoyiannis D (2003). Climate change, the Hurst phenomenon, and hydrological statistics. Hydrol. Sci. J..

[82] Iliopoulou T, Koutsoyiannis D (2019). Revealing hidden persistence in maximum rainfall records. Hydrol. Sci. J..

[83] Serinaldi F, Kilsby C (2016). A blueprint for full collective flood risk estimation: Demonstration for European river flooding: Blueprint for Collective flood risk estimation. Risk Anal..

