# Supplementary material for: On the extreme hydrologic events determinants by means of Beta-Singh-Maddala reparameterization
Source: Sci Rep. 2022 Sep 15;12:15537. doi: 10.1038/s41598-022-19802-4 (PMC9477834; doi:10.1038/s41598-022-19802-4)
Supplement: Supplementary file 1 — Supplementary Information. [file 41598_2022_19802_MOESM1_ESM.pdf]

# On the extreme hydrologic events determinants by means of Beta-Singh-Maddala reparameterization

Filippo Domma[1], Francesca Condino[1], Sara Franceschi[2], Davide Luciano De Luca[3], Daniela Biondi[3]

[1] *Department of Economics, Statistics and Finance "Giovanni Anania", University of Calabria, Arcavacata di Rende, CS, Italy; e-mail: filippo.domma@unical.it, francesca.condino@unical.it*

[2] *Department of Economics and Statistics, University of Siena, Siena, Italy; franceschi2@unisi.it*

[3] *Department of Informatics, Modelling, Electronics and System Engineering, University of Calabria, Arcavacata di Rende, CS, Italy; davide.deluca@unical.it, daniela.biondi@unical.it*

## Appendix

A probability plot was created to check if the Beta-SM4 model provides an adequate fit to the data. The key idea was to find some transformation of the cumulative or (survival) distribution which is linear in some function of the Beta-SM4 random variable. We followed the linearization technique of the survival function described in Lawless (2003, Chapter 18, pp. 99-101).

It is know that if $X$ is a random variable with the following distribution function

$$F_{Beta-SM4}(x;\gamma_1,\gamma_2,\gamma_3,a)=\left[1-(1+\gamma_2 x^{\gamma_3})^{-\gamma_1}\right]^a$$

then $Y=log(X)$ has a log-Beta-SM4 distribution with cumulative function

$$F_{L-Beta-SM4}(y;\gamma_1,\gamma_2,\gamma_3,a)=\left[1-(1+\gamma_2 e^{\gamma_3 y})^{-\gamma_1}\right]^a$$

and $p$th quantile equal to

$$y(p)=\gamma_3^{-1}\left\{\log\left[\left(1-p^{1/a}\right)^{-1/\gamma_1}-1\right]-\log\left(\gamma_2\right)\right\}.$$

Since the moment-generating function of $Y$, $m_y(t)$, can be formulated in terms of the moment of order $t$ of $X$, that is

$$m_Y(t)=E\left(e^{tY}\right)=E\left(e^{t\log(X)}\right)=E\left(X^t\right),$$

the moments of the log-Beta-SM4 distribution can be derived from the moments of the Beta-SM4 distribution reported in Domma and Condino (2016).

It is easy to verify that the cumulative function of the standardized log-Beta-SM4, $Z = (Y - E(Y)) / \sqrt{V(Y)}$, is

$$F_Z(z; \gamma_1, \gamma_2, \gamma_3, a) = \left[1 - \left(1 + \gamma_2 e^{\gamma_3 \left(m + z\sqrt{v}\right)}\right)^{-\gamma 1}\right]^a$$

where $m = E(Y)$ and $v = V(Y)$, while the $q$th quantile turns out to be

$$z(q) = \left(\gamma 3 \sqrt{v}\right)^{-1} \left\{ \log\left[\left(1 - q^{1/a}\right)^{-1/\gamma_1} - 1\right] - \log\left(\gamma_2\right) \right\} - m \left(\sqrt{v}\right)^{-1}.$$

By the above formula, it turns out that

$$z\left(F_{Beta-SM4}(x; \gamma_1, \gamma_2, \gamma_3, a)\right) = \frac{1}{\sqrt{v}} \log\left(x\right) - \frac{m}{\sqrt{v}}$$

is a linear function of $\log(x)$. Therefore, a plot of $z\left(\tilde{F}_{Beta-SM4}(x)\right)$ against $\log(x)$, with

$$z\left(\tilde{F}_{Beta-SM4}(x)\right) = \left(\hat{\gamma}_3 \sqrt{\hat{v}}\right)^{-1} \left\{ \log\left[\left(1 - \tilde{F}_{Beta-SM4}(x)^{1/\hat{a}}\right)^{-1/\hat{\gamma}_1} - 1\right] - \log\left(\hat{\gamma}_2\right) \right\} - \hat{m} \left(\sqrt{\hat{v}}\right)^{-1},$$

where $\tilde{F}_{Beta-SM4}(x)$ denotes the empirical cumulative distribtion function and $\hat{\gamma}_1$, $\hat{\gamma}_2$, $\hat{\gamma}_3$, $\hat{a}$, $\hat{m}$ and $\hat{v}$ are the maximum likelihood estimates of the parameters of the Beta-SM4 distribution, should be roughly linear if the Beta-SM4 distribution is an appropriate model.
